# Correlated high expression of FXR and Sp1 in cancer cells confers a poor prognosis for pancreatic cancer: A study based on TCGA and tissue microarray

**DOI:** 10.18632/oncotarget.16633

**Published:** 2017-03-28

**Authors:** Hai Hu, Lei-Lei Wu, Ting Han, Meng Zhuo, Wang Lei, Jiu-Jie Cui, Feng Jiao, Li-Wei Wang

**Affiliations:** ^1^ Department of Medical Oncology and Pancreatic Cancer Center, Shanghai General Hospital, Shanghai Jiao Tong University School of Medicine, Shanghai 201620, China; ^2^ Shanghai Key Laboratory of Pancreatic Disease, Shanghai 201620, China; ^3^ Department of Medical Oncology and Pancreatic Cancer Center, Renji Hospital, School of Medicine, Shanghai Jiao Tong University, Shanghai 200000, China; ^4^ School of Life Sciences and Biotechnology, Shanghai Jiao Tong University, Shanghai 201620, China

**Keywords:** immunohistochemistry, FXR, Sp1, pancreatic cancer, prognosis

## Abstract

Bile acids (BAs) was critical in the initiation and progression of various tumors. However, their prognostic significance in pancreatic cancer was still illusive. In the present study, the expression and biological significance of FXR, a major receptor of BAs, in the lethal disease were evaluated in mRNA and protein levels. We found that FXR protein was elevated in the cancerous tissues, which was significantly higher than the adjacent tissues (*p* < 0.05). Meanwhile, our data showed that FXR was positively correlated with primary tumor location (*p* = 0.04) and poor survival (*p* = 0.002). Finally, COX regression model indicated that FXR protein was an independent prognostic factor (*p* = 0.01; HR = 2.15; 95% CI 1.27-3.63). Consistently, we also found a significant difference of FXR expression between the high and low groups in mRNA level (*p* < 0.001), and that high FXR expression confers a poor prognosis (*p* < 0.001). More importantly, the correlation assay showed that FXR was positively correlated Sp1 in both protein (*r* = 0.351, p = 0.008) and mRNA levels (*r* = 0.263, *p* < 0.01), with the simultaneously high expression indicated the worst prognosis on protein (*p* < 0.001) and mRNA levels (*p* < 0.001). Additionally, we also showed that FXR was elevated in the pancreatic cancer cells responsible for proliferation and migration. Overall, the data suggested co-high expression of the two factors was an independent prognostic factor (*p* < 0.001; HR = 3.27; 95% CI 1.86–5.76). Based on these data, we proposed a model to link FXR to Sp1, which included triggered FXR, p38/MAPK and/or PI3K/AKT signaling and phosphorylated Sp1, to illustrate the potential crosstalk between the two factors.

## INTRODUCTION

Pancreatic cancer is a lethal disease with the 5-year survival rate of less than 5% and the median survival of 6 months, rendering it the fourth cause of cancer-related deaths [1]. In practical settings, only 15% patients were diagnosed at early stages and suitable for radical resection, which offers the only chance for cure [2]. On the contrary, most patients were diagnosed as metastatic disease, for which chemotherapy was their only option. However, drugs available only bring modest benefits dues to chemoresistence [3–5]. Hence, it is of great urgency to identify new therapeutic targets so as to improve the prognosis for the patients.

Bile acids (BAs) are natural end products of cholesterol metabolism that function to emulsify and solubilize the lipid aggregates. In the recent years, much had learned between BAs and tumorigenesis, and nearly all studies suggested that BAs involved extensively in human cancer initiation and progression [6, 7]. Farnesoid X receptor (FXR) was a key receptor of BAs, and it was overexpression in various human malignancies associating with anti- and/or pro-tumoral roles. For instance, prior studies indicated that inactivation of FXR in cancer of liver and breast corresponded with increased propagation and decreased apoptosis [8, 9]. While, a study by B Guan and colleagues showed the opposite, they found that FXR inhibition limits the growth of esophageal cancer [10]. As to pancreatic cancer, the existing studies had concluded an overexpression profile of FXR with conflicting roles. For instance, Lee JY showed that FXR functions as oncogene since it positively correlated with lymph node metastasis, migration and invasion of pancreatic cancer [11]; while another direct evidence, obtained more recently, indicated that elevated FXR in pancreatic cancer was responsible for a less aggressiveness phenotype and favors a better prognosis [12]. Obviously, the conflicting results promoted us to re-evaluate the function of FXR in the lethal disease.

In the present study, we examined the expression and biological significance of FXR in pancreatic cancer in a larger population from both protein and mRNA levels. We showed that FXR was upregulated in both mRNA and protein levels, and its overexpression confers a poor prognosis for the patients. In addition, we also showed that FXR was positively correlated with Sp1, a factor found to be an independent prognostic factor for pancreatic cancer in our prior study [13]. Their co-high expression confers the poorest prognosis among all the patients, which could regard as an independent prognostic factor for the patients. Finally, we proposed a model, which included activated FXR signaling to Sp1, to illustrate the detailed crosstalk between Sp1 and FXR.

## RESULTS

### Correlated Sp1 and FXR expression in mRNA level in pancreatic cancer tissues

**Figure 1:**
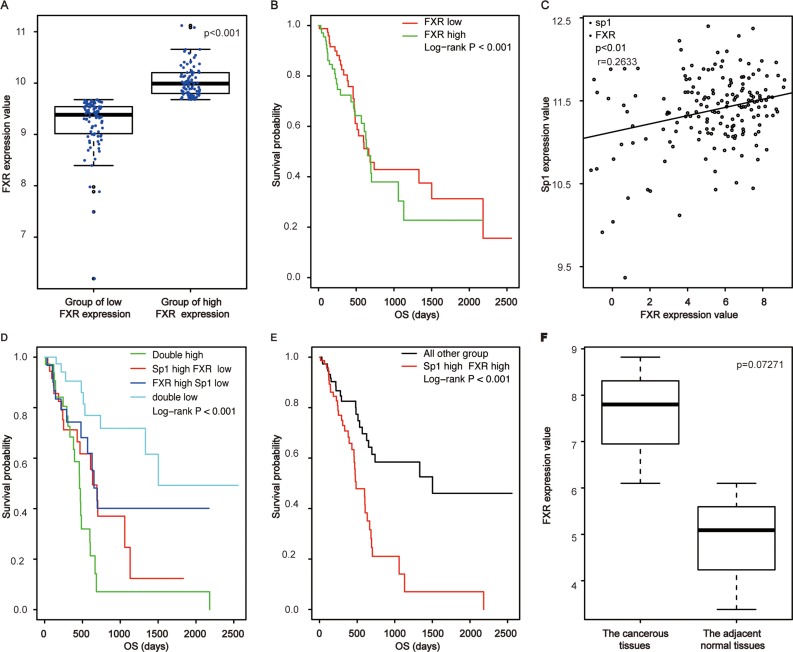
Correlated high expression of Sp1 and FXR in mRNA levels confers the poorest prognosis for pancreatic cancer (**A**) The comparison of FXR expression between the high group and the low one; (**B**) Survival analysis of pancreatic cancer patients based on FXR expression; (**C**) Correlation assay between FXR and Sp1 in mRNA level; (**D**, **E**) Survival analysis of pancreatic cancer patients based on Sp1 and FXR in mRNA level; (**F**) The comparison of FXR expression between the cancerous tissues and the paired none cancerous tissues in mRNA level.

To investigate the role of FXR in pancreatic cancer; we initially examined its expression in mRNA level in clinical samples of pancreatic cancer. To this end, the RNA-seq data of pancreatic cancer was downloaded from TCGA. We found that FXR expressed ubiquitously with different levels (Figure 1A). Moreover, a significant difference could be observed between high and low expression groups (*p* < 0.001, Figure 1A). Furthermore, survival analysis showed that high FXR expression indicated a poor prognosis for the patients (Table 1, *p* < 0.001, Figure 1B).

**Table 1:** The detailed survival data of pancreatic cancer based on FXR expression

		Number at risk		
Year	0 (year)	2 (year)	4 (year)	6 (year)
Low	87(49.7%)	16(9.1%)	6(3.4%)	1(0.6%)
High	88(50.3%)	9(5.1%)	3(1.7%)	0(0.0%)
	Median survival time (year)			
Low	1.83			
High	1.78			
Survival rate	Three year survival rate (95% CI)		Five year survival rate	
Low	0.4288(0.3020~0.6089)		0.3127(0.1776~0.5503)	
High	0.3036(0.1646~0.5600)		0.2277(0.0989~0.5241)	

Specificity protein (Sp1) is a nuclear transcription factor locating in the nuclear. It expressed ubiquitously in all cells of an individual and responsible for proliferation, differentiation and apoptosis [14, 15]. We found previously that elevated Sp1 confers a poor prognosis for pancreatic cancer [13]. Since activated FXR could trigger line of signaling associating with cell proliferation, metastasis and chemoresistence by phosphorylating downstream molecule, and phosphorylated Sp1 was the active form of Sp1 [16]; we postulated that the two factors were related in pancreatic cancer. Base on the hypothesis, we examined their correlationship and found that they were positively correlated (*p* < 0.01, r = 0.263, Figure 1C) in mRNA levels with their simultaneously high expression indicated the poorest prognosis for the patients (*p* < 0.001, Figure 1D, 1E). Of note, we showed no significant differences of FXR expression between the cancerous tissues and the paired adjacent tissues (*p* = 0.0727, Figure 1F), which might cause by the small samples of adjacent tissues included in TCGA.

### FXR was overexpression in protein level in pancreatic cancer tissues

**Figure 2:**
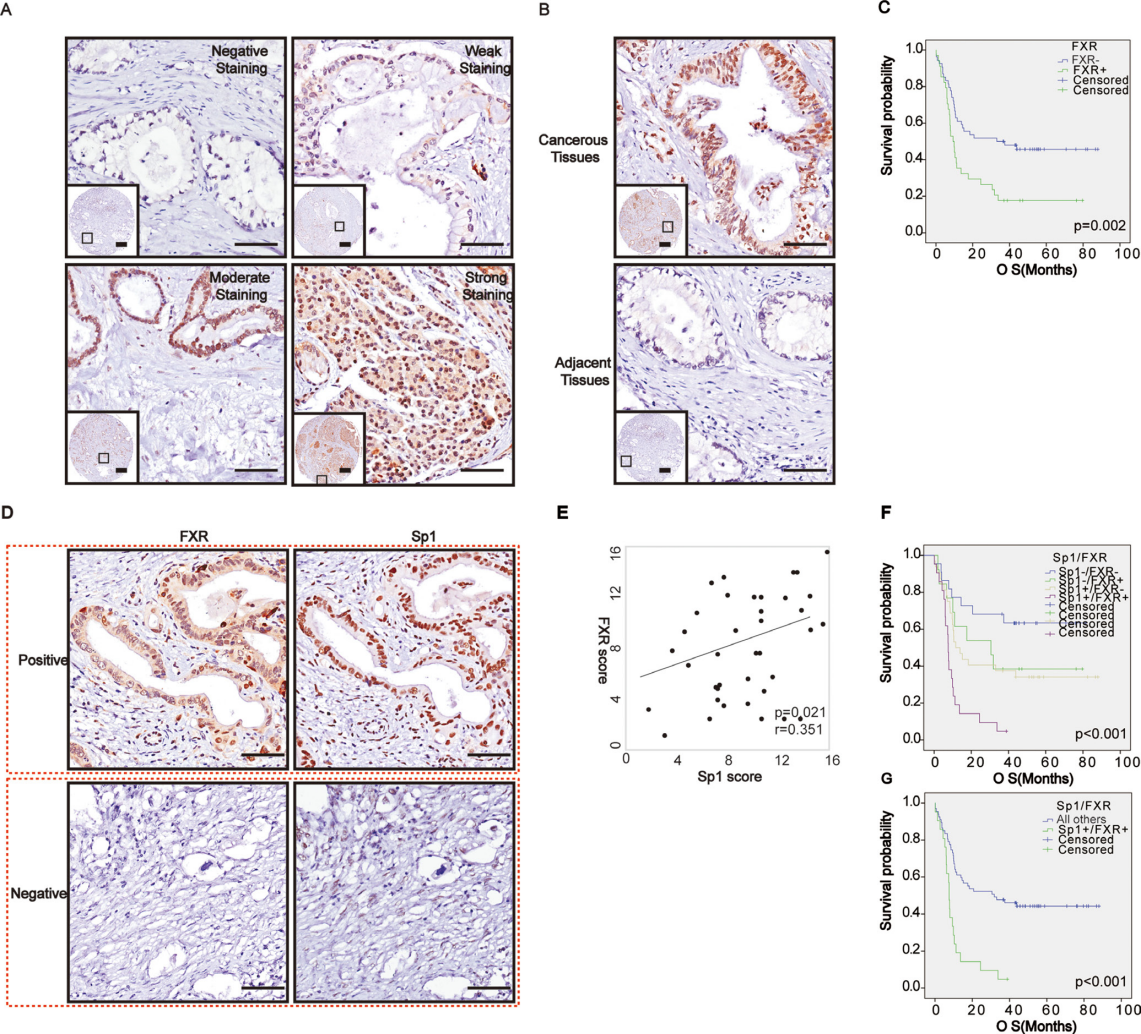
Correlated high expression of Sp1 and FXR in protein levels confers the poorest prognosis for pancreatic cancer (**A**) Representative imagines of FXR staining in pancreatic cancer; (**B**) Representative imagines of FXR staining in the cancerous tissues and paired normal tissues; (**C**) Survival analysis based on FXR expression; (**D**) Representative imagines depicted the correlation between Sp1 and FXR in the series sections; (**E**) Correlation assay between Sp1 and FXR staining in pancreatic cancer; (**F**–**G**) Survival analysis based on FXR and Sp1 expression. All others: Sp1^+^FXR^−^, Sp1^−^FXR^+^, and Sp1^−^FXR^−^.

To confirm the role of FXR in pancreatic cancer; we then examined its expression in protein level by immunohistochemistry. We showed that patients exhibited different levels of FXR expression ranging from negative to strong positive (Figure 2A). In addition, our data also showed that there was a significant difference of FXR expression between the cancerous tissues and the paired normal tissues (*p* < 0.05, Table 2, Figure 2B). Moreover, Pearson's chi-squared test showed that high FXR expression was positively correlated with tumor location (*p* = 0.04, Table 3), but not with other parameters of the patients, such as age, gender and so on. Finally, Kaplan–Meier analysis suggested that high FXR expression confers a poor prognosis (*p* = 0.002, Figure 2C), and its high expression could be regarded as an independent prognostic factor (*p* < 0.001; HR = 3.27; 95% CI 1.86–5.76, Table 4).

**Table 2:** FXR expression in the cancerous tissues and the adjacent tissues

	Number	FXR		*P*
		Positive	Negative	
Cancerous tissues	88	54 (61.4%)	34 (38.6%)	***P* < 0.05**
Adjacent tissues	88	27 (30.7%)	61 (69.3%)	

**Table 3:** Correlation between FXR expression and the clinicopathologicfeatures of pancreatic cancer patients

Factor	*N*	FXR		
		Positive	Negative	*P*
Gender				
Male	56 (63.6%)	34 (38.6%)	22 (25%)	0.87
Female	32 (36.4%)	20 (22.7%)	12 (13.7%)	
Age				
≤ 60	41 (46.6%)	24 (27.3%)	17 (19.3%)	0.61
> 60	47 (53.4%)	30 (34.1%)	17 (19.3%)	
Tumor stage				
≤ T2	74 (84.1%)	45 (51.1%)	29 (33%)	0.81
T3	14 (15.9%)	9 (10.2%)	5 (5.7%)	
Nodal stage				
N0	52 (59.1%)	32 (36.3%)	20 (22.7%)	0.97
N1	36 (40.9%)	22 (25%)	14 (15.9%)	
Primary tumor location				
Body and Tail	30 (34.1%)	14 (15.9%)	16 (18.2%)	0.04
Head and Neck	58 (65.9%)	40 (45.5%)	18 (20.4%)	
Lymphvascular invasion				
No	50 (56.8%)	32 (36.4%)	18 (20.5%)	0.56
Yes	38 (43.2%)	22 (25%)	16 (18.2%)	
Nuclear grade				
≤ II	73 (83%)	48 (54.5%)	25 (28.4%)	0.06
> II	15 (17%)	6 (6.8%)	9 (10.2%)	
Jaundice				
No	62 (70.5%)	36 (40.9%)	26 (29.6%)	0.33
Yes	26 (29.5%)	18 (20.5%)	8 (9%)	
Abdominal pain				
No	39 (44.3%)	27 (30.7%)	12 (13.6%)	0.18
Yes	49 (55.7%)	27 (30.7%)	22 (25%)	

**Table 4:** Univariate and multivariate survival analysis of clinic-pathologic variables of pancreatic cancer patients

Factor	OS (Months) Median (range)	Univariate analysis			Multivariate analysis		
		HR	95%CI	*P*	HR	95%CI	*P*
Gender							
Male	11.1 (0.2–88.0)	0.58	0.33–1.03	0.07			
Female	36.7 (0.1–76.4)	1					
Age							
> 60	11.3 (0.2–86.7)	1.05	0.63–1.77	0.84			
≤ 60	15.2 (0.1–88.0)	1					
T stage							
T3	23.5 (0.2–88.0)	1.03	0.50–2.09	0.95			
≤ T2	12.6 (0.1–86.7)	1					
Nodal stage							
N0	33.5 (0.2–88.0)	0.69	0.53–0.90	0.006	0.55	0.31–0.95	0.03
N1	9.8 (0.1–86.7)	1			1		
Primary tumor location							
Head/Neck	14.8 (0.1–88.0)	0.83	0.49–1.43	0.51			
Body/Tail	10.5 (1.3–79.6)	1					
Lymphvascular invasion							
Yes	10.6 (0.2–82.4)	1.39	0.82–2.33	0.22			
No	18.0 (0.1–88.0)	1					
Nuclear grade							
≤ II	18.4 (0.2–88.0)	0.68	0.49–0.93	0.02	0.46	0.23–0.91	0.03
> II	7.0 (0.1–79.6)	1			1		
Jaundice							
No	14.2 (0.2–82.4)	0.98	0.55–1.74	0.94			
Yes	11.5 (0.1–88.0)	1					
Abdominal pain							
No	10.6 (0.1–88.0)	0.71	0.42–1.19	0.19			
Yes	17.6 (2.7–86.7)	1					
Sp1							
Positive	9.9 (0.1–88.0)	2.42	1.35–4.33	0.003	2.27	1.24–4.16	0.008
Negative	37.4 (0.2–81.5)	1			1		
FXR							
Negative	9.6 (0.1–79.6)	2.15	1.27–3.63	0.004	2.02	1.16–3.49	0.01
Positive	34.8 (0.2–88.0)	1			1		
Sp1/FXR							
Sp1+/FXR+	10.1 (0.2–88.0)	3.27	1.86–5.76	< 0.001	2.71	1.53–4.80	0.001
All others	34.2 (0.1–82.4)	1			1		

### Correlated Sp1 and FXR expression in protein level in pancreatic cancer tissues and cell lines

Subsequently, we tried to investigate whether the correlation between Sp1 and FXR also exists in pancreatic cancer in protein level. To obtain this, we observed their expression in the serial sections of tissue microarray and found that where FXR staining accompanied by Sp1 staining, and vice versa (Figure 2D). Statistically, Spearman's rank test also revealed a positively correlation between them (*p* = 0.021, r = 0.35, Table 5, Figure 2E). Furthermore, survival assay indicated that their simultaneous high expression confers the poorest prognosis among all the patients (*p* < 0.001, Figure 2F, 2G), and their combined high expression was also an independent prognostic factor for the patients (*p* < 0.001, HR = 3.27, 95% CI 1.86-5.76, Table 4).

**Table 5:** FXR was positively correlated with Sp1 in protein level in pancreatic cancer

Parameters		FXR			χ^2^	Co-efficient	*P*
		Positive	Negative	Total			
**Sp1**	**Positive**	40 (45.5%)	13 (14.8%)	53 (60.2%)	11.19	0.35	*P* < 0.05
	**Negative**	14 (15.9%)	21 (23.9%)	35 (39.8%)			
	**Total**	54 (61.4%)	34 (38.6%)	88 (100%)			

**Figure 3:**
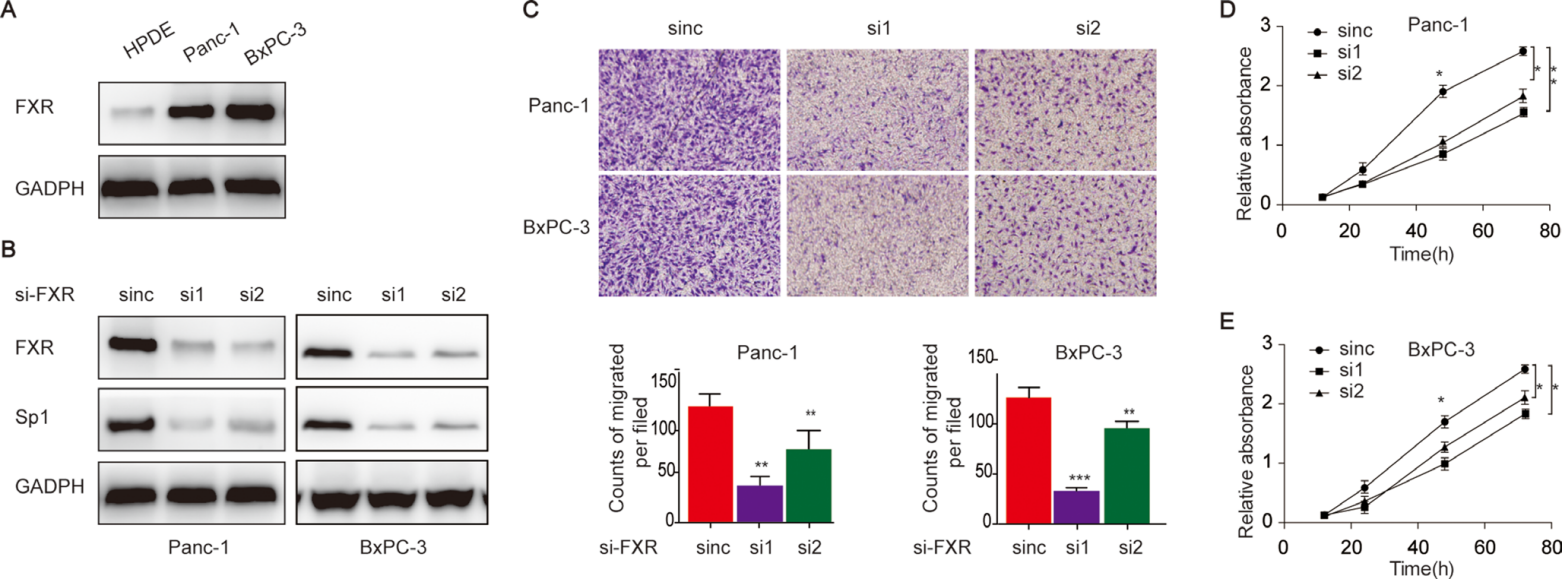
Elevated FXR in pancreatic cancer cells associate with increased proliferation and migration capacities (**A**) FXR expression in pancreatic cancer cell line and HPDE; (**B**) The detection of Sp1 expression upon FXR knockdown; (D/E) The evaluation of proliferation (**C**) the evaluation of pancreatic cancer proliferation after FXR knockdown; (**D**) and migration (**E**) of pancreatic cancer cells upon FXR knockdown.

Meanwhile, we also investigated whether the positive correlation exists in the cell line of pancreatic cancer in protein level. To begin with, we detected FXR expression in the cell lines. We found that FXR expression was higher in the cancerous cell lines when compared to the normal cell- human pancreatic duct epithelial (HPDE) cells (Figure 3A). After that, we investigated Sp1 expression after FXR deletion in the cancer cell lines so as to reveal their correlation. The data indicated that Sp1 expression was down regulated upon FXR knockdown (Figure 3B), suggesting that the positive correlation also existed in pancreatic cancer cells in protein level.

Additionally, the biological behaviours of pancreatic cancer cells were examined upon FXR knockdown. We showed that the cells showed decreased migration upon FXR knockdown (Figure 3C). Consistently, our data also indicated that the cells showed decreased proliferation upon FXR knockdown (Figure 2D, 2E). Taken together, these data indicated that elevated FXR expression was a risk factor pancreatic cancer initiation and progression.

### Proposed model linking FXR to Sp1 in pancreatic cancer

**Figure 4:**
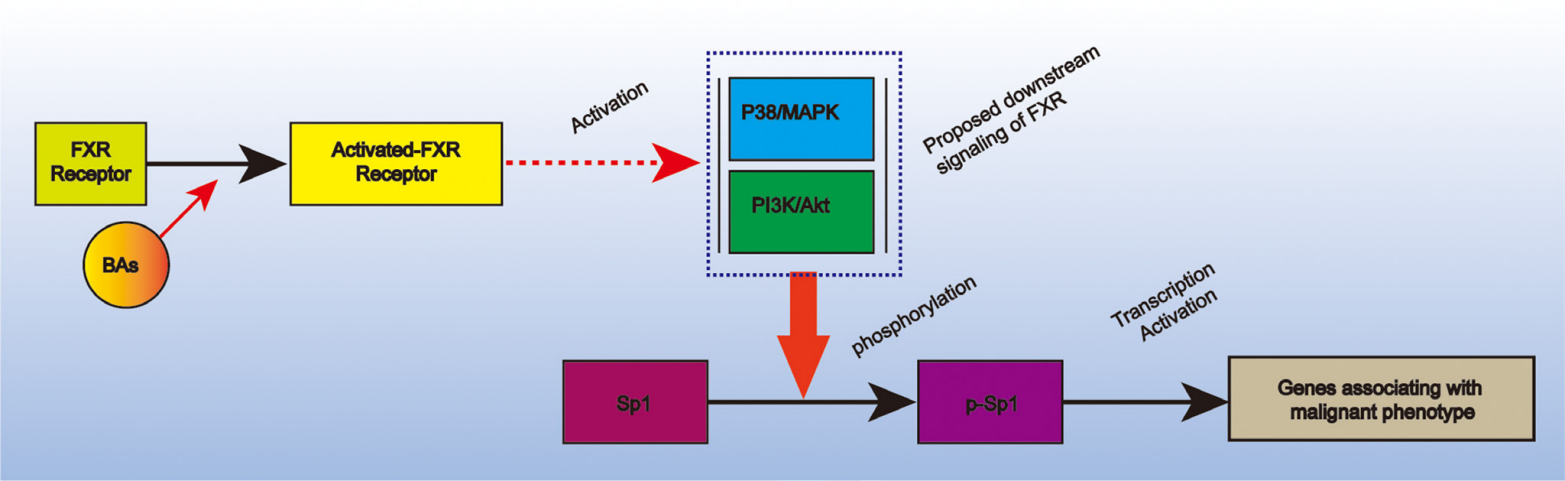
Proposed model that link Sp1 and FXR in pancreatic cancer It depicted that BAs-triggered FXR could phosphorylate Sp1 via p38-MAPK and/or PI3K-AKT signaling. The activated Sp1 could subsequently promote pancreatic cancer progression via transcription activation.

Systematical literature review indicted that FXR functions through activating downstream signaling, such as p38/MAPK [17] and PI3K/AKT [18], which subsequently phosphorylate downstream molecule, leading to deregulated cell proliferation, invasion and metastasis, and chemoresistence. This very fact in combined with our finding that FXR was positively correlated Sp1 and the discovery that phosphorylated Sp1 was the activated form of Sp1 promoted us to proposed a model to link FXR to Sp1: BAs-triggered FXR increase the over-activation of p38-MAPK and/or PI3K/AKT signaling in the plasma resulting in the phosphorylation of Sp1, which finally activate the expression of genes associating with the malignant phenotype of pancreatic cancer (Figure 4).

## DISCUSSION

In the present study, FXR expression and its biological significance in pancreatic cancer were investigated. We showed that FXR expressed ubiquitously in pancreatic cancer, and that high FXR expression confers a poor prognosis. Moreover, we also found that Sp1 was positively correlated with FXR with their simultaneous high expression confers the poorest prognosis for the patients. Based on the data, we proposed a model to illustrate the crosstalk between FXR and Sp1. Taken together; our study added the wealth knowledge on the pro-tumoral role of FXR in pancreatic cancer and suggested that targeted inhibition of FXR and/or in combination of Sp1 might provide an alternative approach for the management of pancreatic cancer.

Bile acids (BAs) are natural end products of cholesterol metabolism that function to emulsify and solubilize the lipid aggregates. Previously, researches on BAs were mainly focused on its role in chronic inflammation, such as acute pancreatitis [19] and gastritis [20, 21], and the positive correlation between chronic inflammations of the gastrointestinal tract organs had now been well established. Since inflammation was a widely accepted risk factor for human malignancies [22, 23], mounting studies were conducted to investigate the role of BAs on tumorigenesis in the recent years. Most of the studies concluded BAs to be risk factor for human cancers, especially for the gastrointestinal tract ones [6], despite some of the existing data showed that BAs to be an anti-tumoral factor [24]. As to pancreas, none systematically investigated the role of BAs in pancreatic carcinogenesis. Recently, a study completed by Tomohiko Adachi showed that refluxed BAs into pancreatic ducts to be a significant factor predisposing to pancreatic intraductal papillary carcinomas [25], which suggested that BAs might also function as a pro-tumoral factor in pancreatic cancer.

FXR is a nuclear receptor that responsible for bile acid homeostasis via regulating the expression of genes involved in the process [26, 27]. However, there were various reports that FXR was elevated in multiple human cancers responsible for their initiation and progression [28, 29]. For instance, it is stated that FXR could suppress the proliferation of liver cancer via the inhibition of mTOR/S6K signaling [8]. On the other hand, some of the reports proposed the opposite; they showed that that the inhibition of FXR limits esophageal cancer growth [10]. The paradox data indicated that the exact role of FXR was tissue-specific. As to pancreatic cancer, the reported function of FXR was also paradox, with Giaginis C showed that FXR was anti-tumoral [12], while Lee JY showed that FXR overexpression facilitates lymphatic metastasis of pancreatic cancer [11]. Apparently, our data contradicted with Giaginis C. Comprehensive review of these studies showed that our data was more convincible, as we included a larger sample size, and the conclusion were drawn from both the mRNA and protein levels.

Considering the biological significance, we preliminary investigated the machinery whereby FXR interacts with Sp1 in pancreatic cancer. Since FXR could functions via phosphorylating downstream molecule [16], and phosphorylated Sp1 was the active form of Sp1 [30]. We hence hypothesized that Sp1 functions downstream of FXR. As Sp1 was a transcription factor, there was possibility that elevated FXR in pancreatic cancer resultant from elevated Sp1 transcription activity. Indeed, this might give the explanation of the correlated Sp1 and FXR in the disease. Overall, these hypotheses were merely based on literatures review and needed further experimental confirmation.

In conclusion, our findings reveal strong expression of FXR in pancreatic cancer, and suggested that FXR may serve as an oncogenic factor that promotes pancreatic progression by collaborating with Sp1. Since both FXR and Sp1 were associated with the malignant phenotypes of pancreatic cancer, further study is required to determine their potential roles to be candidate therapeutic target in the clinics.

## MATERIALS AND METHODS

### Bioinformatics analysis

RNA-Seq data of PDAC was downloaded from TCGA (http://cancergenome.nih.gov/). The data includes 183 pancreatic cancer patients in total, and four patients were with paired-none cancerous tissues. Other information of the patients, such as age, gender, prognosis and so on, were obtained from the website. For statistical analysis, the patients were divided into halves base on Sp1 and FXR expression values. Specifically, patients with the expression values greater than the median value were classified into the high expression group, while the rest were added to the low expression group.

### Cell lines and cell culture

Human pancreatic duct epithelial (HPDE) cells and pancreatic cancer cell lines were purchased from Shanghai Institute for Life Science, Chinese Academy of Sciences. All the cells were cultured in RPMI 1640 supplemented with 10% fetal bovine serum (FBS, Gibco, Carlsbad, CA, USA) at 37°C in a humidified atmosphere of 95% air and 5% CO2, and grown in a humidified atmosphere of air/CO2 (95%: 5%). Cells with gene deletion were cultured in the same condition with 1.5-μg/mL puromycin (Sigma-Aldrich, St. Louis, MO, USA). For passage and experimental purposes, the cells were detached using trypsin-EDTA and resuspended in the complete medium.

### siRNA transfections

Cells were plated in 6-wells plates and transfected with 3 μl RNAi in the presence of 4μl RNAimax (Invitrogen) according to manufactures’ instructions. Two different siRNAs and the control siRNA were purchased from Genepharm Technologies (Shanghai, China). Gene silencing effects were confirmed by Western blot analysis at 48 hours post-transfection. Specific siRNA oligo duplexes targeting FXR (FXR siRNA#1:5′-GCGGTTGAAGCTATGTTCCTTCGTT-3′;FXR siRNA# 2:5′-GGCTCCAGGGAATCCTGCATTCTAA-3′) and the negative control siRNA were synthesized by GenePharma (Shanghai, China).

### Migration assays

For migration assays, the treated cells were grown in 60-mm plates in RPMI-1640 without growth factors for 24h. The complete media were added to the bottom chambers of 24-well tissue culture plates in triplicate. The cells (40,000) were added to the upper chambers of Transwell assays (BD Biosciences, Franklin) Cells were allowed to migrate for 14 h and then fixed, stained, and quantified.

### Cell proliferation CCK8 assay

For *in vitro* proliferation cells were plated at 1000 cells per well of a 96 well plate and after 16 h, growth medium with different concentrations of the respective FXR agonist was added. After 72 hours, the DNA content was determined using a spectro-fluorometer (Envision, Perkin Elmer, Boston, U.S.A.) using the CyQuant Direct Proliferation Assay (according to the manufacturers recommendations.

### Western blot analysis

Cells were washed three times with cold PBS and lysed on ice in RIPA buffer with protease inhibitors PMSF (Beyotime Biotechnology, China). Protein concentrations were determined by BCA method (Beyotime Biotechnology, China). A total of 20 μg protein was separated by 10% SDS-PAGE and electro-blotted onto NC membranes using semi-dry blotting apparatus. After blocking in 3% bovine serum albumin (BSA), the membranes were incubated with the primary antibodies overnight at 4°C. The membranes were washed and incubated with the secondary antibodies for 1h at room temperature on a shaker. The protein bands were visualized using a commercially available enhanced chemiluminesence kit (Thermo Scientific, Hudson, NH, USA). GAPDH were used as control. The primary antibodies used in the study include: Sp1(1:1000), FXR:1(1000) (CST, Beverly, MA, USA); and GAPDH (Santa Cruz Biotechnology, CA, USA)

### Patients

88 patients in total who were histopathologically diagnosed as pancreatic cancer at the Department of General Surgery, Shanghai General Hospital affiliated Shanghai Jiao Tong University from 2009 to 2012. The histological characterization and clinic pathological staging of the samples were determined according to current International Union Against Cancer Guidelines. Cancerous and adjacent normal tissues were collected from patients during the surgeries. Written informed consents and approvals from the Ethics Committees of Shanghai General Hospital were obtained for the use of these materials for research purposes. The study was consistent with the provisions of the Declaration of Helsinki (as revised in Fortaleza, Brazil, October 2013).

### Tissue microarray construction

The microarray was made in collaboration with Shanghai Biochip, Shanghai, China. Briefly, hematoxylin and eosin (HE) stained sections from primary tumor blocks were used to define two representative tumor regions and adjacent normal tissues: representative tumor regions were defined as tumor areas containing more than 75% cancer cells without necrosis; and adjacent normal tissues at least 5 cm from the tumor regions were randomly selected. Samples were not taken from areas of bleeding. Cylinders (1.5 mm in diameter) were then punched from defined regions of a tissue block using a tissue microarrayer (Century, IL, CA, USA) and inserted into recipient paraffin blocks. Two sets of three paraffin-embedded tissue microarray blocks were made, and sections of these blocks were transferred onto glass slides. In total, two sets of tissue microarray blocks containing 88 tumor tissue spots and 88 adjacent normal tissue spots were used in this study.

### Immunohistochemistry

A standard immunohistochemistry protocol was used as previously described [31]. Briefly, tissue microarray sections were dewaxed and dehydrated in a xylene and alcohol bath solution. Endogenous peroxidase activity was then blocked by a 10-min incubation in 0.3% hydrogen peroxide. Antigen retrieval was then achieved by incubating the slides in 0.01 M citrate buffer (pH 6.0) at 98°C for 5 min using a microwave oven. The slides were then cooled to room temperature and blocked in normal goat serum at room temperature for 1 h, followed by incubation with a primary antibody at 4°C overnight. The sections were then incubated with a horseradish peroxidase-labeled secondary antibody and visualized using 3,3′-diaminobenzidine.

### Score of staining density

Evaluation of the staining in at least five areas at 400 × magnification was performed by two independent pathologists blind to study. The staining was scored according to the intensity and percentage of the stained cells. Staining intensity was assigned as 1 (no staining), 2 (weak staining), 3 (moderate staining), and 4 (strong staining). The percentages were classified into four categories: 1 (≤ 25%), 2 (25%–50%), 3 (50%–75%), and 4 (75%–100%). The final scores were calculated as the staining intensity × the percentage of positive cells. For statistical analyses, a score < 8 was regarded as negative expression, and > 8 as positive expression.

### Statistical analysis

Statistical analyses were performed using SPSS (version 21.0; SPSS Inc., Chicago, USA). The relationships between the clinicopathlogical factors and FXR were investigated using Pearson χ^2^ test. The Spearman's rank test was used to evaluate the correlation between Sp1 and FXR. Kaplan–Meier analysis was used to demonstrate differences in overall survival (OS). The correlation between the factors and OS were investigated with the Cox regression model. Factors correlating with OS in the univariate analysis were tested by multivariate analysis. The hazard ratio (HR) and corresponding 95% confidence intervals (95% CI) were calculated for each factor. Data were considered statistically significant when *p* < 0.05.
